# Trends in workplace violence for health care occupations and facilities over the last 10 years

**DOI:** 10.1093/haschl/qxae134

**Published:** 2024-10-23

**Authors:** Brianna Lombardi, Todd Jensen, Evan Galloway, Erin Fraher

**Affiliations:** Carolina Health Workforce Research Center, Cecil G. Sheps Center for Health Services Research, University of North Carolina at Chapel Hill, Chapel Hill, NC 27516, United States; Department of Family Medicine, School of Medicine, University of North Carolina at Chapel Hill, Chapel Hill, NC 27516, United States; School of Social Work, University of North Carolina at Chapel Hill, Chapel Hill, NC 27516, United States; School of Education, University of North Carolina at Chapel Hill, Chapel Hill, NC 27516, United States; Carolina Health Workforce Research Center, Cecil G. Sheps Center for Health Services Research, University of North Carolina at Chapel Hill, Chapel Hill, NC 27516, United States; Carolina Health Workforce Research Center, Cecil G. Sheps Center for Health Services Research, University of North Carolina at Chapel Hill, Chapel Hill, NC 27516, United States; Department of Family Medicine, School of Medicine, University of North Carolina at Chapel Hill, Chapel Hill, NC 27516, United States

**Keywords:** workplace violence, health care occupations, health care facilities, health workforce

## Abstract

Issues of workplace violence (WPV) in health care have garnered increasing attention due to the impact on the health care worker’s well-being and retention. Yet, our understanding of whether and how WPV rates vary between health care facilities and occupations is limited, particularly information on growth over time. This information is needed to develop and target policies and interventions toward health care workers and settings most at risk. We examined trends in WPV among health care occupations and facilities over the past decade (2011–2021/2022), utilizing data from the Bureau of Labor Statistics’ Survey of Occupational Injuries and Illness. Findings reveal a 30% increase in WPV across all health care facility types between 2011 and 2021/2022; however, there was no difference in the average rate of WPV for health care occupations over the same time period. The increase in WPV for health care facilities began long before the pandemic, suggesting larger systemic issues are likely driving WPV. Existing state and organizational efforts aim to mitigate WPV, yet targeted interventions are crucial. Understanding variations across occupations and facilities will inform tailored strategies to safeguard health care workers.

## Introduction

Health systems, professional organizations, and news reports suggest that workplace violence (WPV) has increased since the start of the COVID-19 pandemic, adding to the immense pressure facing the health workforce.^1-5^ The consequences of WPV extend beyond immediate physical harm, encompassing psychological distress, lower worker well-being, decreased job satisfaction, increased turnover rates, and compromised patient care quality.^6-9^ Workplace violence is defined by the Occupational Safety and Health Administration (OSHA) as “any act or threat of physical violence, harassment, intimidation, or other threatening disruptive behavior that occurs at the work site.”^10^ Addressing WPV is critical because it affects recruitment and retention of health care workers. Health settings and professions that have high WPV rates may struggle to recruit individuals and retain them once they are in the workforce. At the same time, health systems are working to address WPV through prevention and remediation strategies, including data collection and reporting on WPV incidents, risk-assessment strategies, trauma support, and efforts to create a culture of workplace safety. Many states have passed legislation to protect health care workers, including assault penalties against individuals perpetrating violence and requirements for organizations to have preventative and post-incident response measures in place.

Workplace violence in health care is not a new phenomenon related to the pandemic; according to the US Bureau of Labor Statistics (BLS), intentional violence toward health care workers increased 63% between 2011 and 2018.^11^ Relative to other sectors, health care professionals face a disproportionate risk of experiencing violence at work: 73% of all nonfatal injuries due to violence that occurred in a US work setting was experienced by health care workers.^11^ In 2021/2022, US health care practitioners experienced 7.8 WPV events per 10 000 workers and health care support occupations experienced 13.6 events per 10 000 workers.^11^ Data from surveys of health occupations have found similarly high rates of WPV: 61% of home health care workers, 44% of nurses, and 21% of emergency department physicians report experiencing physical assaults from patients.^12-14^ Evidence suggests that health care occupations employed in some settings have higher WPV rates, including workers in emergency departments, psychiatric facilities, and home health care settings.^15,16^ Yet, it is unclear if the rates of WPV are increasing at similar rates across all health care occupations and facilities or if they vary by setting or occupation.

Despite growing recognition of the problem and increasing concern that WPV is driving workers to exit the health workforce, our understanding of whether and how WPV rates vary between health care facilities and occupations is limited. This gap is important because, beyond documenting the overall incidence and prevalence of WPV against health care workers, we lack a more nuanced understanding of WPV needed to develop and target policies and interventions toward health care workers and settings most at risk. The underlying factors contributing to WPV rates likely differ between occupations and settings and policymakers, health system leaders, employers, and others would benefit from data to inform their investments to prevent and address WPV. This study investigates trends in WPV for different types of health care workers and across different health care facilities over a 10-year period from 2011–2021/2022.

## Data and methods

### Data source and sample

This study used data drawn from the BLS’ Survey of Occupational Injuries and Illness (SOII). The SOII contains employer-reported information on illnesses and injuries that occur within an employment-based setting in the United States. The SOII is a survey of approximately 200,000 employers annually. Employers are asked to provide data from workplace injury logs that they are required to maintain by OSHA. The BLS requests that employers report total workplace injuries, and then asks for additional detail about injuries resulting in days away from work (DAFW). The SOII also asks for employee hours, which enables rates of injury and illness incidents to be reported and calculated. The SOII data are publicly available and annualized per 10 000 full-time workers.

In this study, we assessed trends in nonfatal workplace injuries that resulted in at least 1 DAFW related to the event code for “violence and other injuries by persons.” Beginning in 2022, the SOII began releasing data on a biannual basis to allow for larger sample sizes that would permit public reporting of more detailed information on illnesses and injuries. Prior to 2021–2022, the SOII was reported annually. This study used the BLS SOII–calculated rates of WPV events per 10,000 FTE (full-time equivalents) for detailed occupations and industries. Files for analyses are publicly available online.^17,18^

The SOII captures data for occupations using the Standard Occupational Classification (SOC) system and for setting or facility type using the North American Industry Classification System (NAICS). The SOC includes 2 primary categorizations of health care workers: Healthcare Practitioners and Technical Occupations (29-0000) and Healthcare Support Occupations (31-0000). We included 45 SOC codes that were included in the 29-000 and 31-0000 SOC categories, which also included any yearly data prior on WPV prior to 2021/2022. We included the 17 NAICS 4-digit codes that are related to health settings that fall within the Health Care and Social Assistance Industry classification (see Table 1 for a list of included facilities: eg, offices of physicians [except mental health specialists]; offices of physicians, mental health specialists; offices of other health practitioners; outpatient care centers; general medical and surgical hospitals). Occupations included from the SOC and settings included from the NAICS were mutually exclusive categories.

**Table 1.** Rate of workplace violence incidents per 10,000 full-time workers within selected health care occupations and industries (2011–2021/2022).

Health care and social assistance industry classification	2011	2012	2013	2014	2015	2016	2017	2018	2019	2020	2021/2022
Offices of physicians (except mental health specialists)	0.3	0.1	0.3	0.3	0.4	0.2		0.8	0.5	0.4	0.8
Offices of physicians, mental health specialists		17.3		8.5			6.1		26.6	6.0	8.7
Offices of other health practitioners		1.6	0.9		1.3	1.9	2.6		10.5	2.7	4.0
Outpatient care centers	2.5	4.7	4.2	4.1	3.2	2.9	3.1	3.9	4.8	5.2	2.9
Medical and diagnostic laboratories				5.6			7.1	0.6			
Home health care services	3.0	2.8	3.8	5.0	4.1	4.6	6.6	4.1	4.1	5.0	2.9
Other ambulatory health care services	2.2	1.9	1.5	3.1	3.4	2.4	2.7	2.0	5.4	9.2	3.8
General medical and surgical hospitals	5.0	5.8	6.8	6.7	6.6	7.2	8.0	9.7	9.4	14.3	12.9
Psychiatric and substance abuse hospitals	64.5	69.6	85.1	109.5	84.6	82.7	121.1	124.9	107.5	114.2	110.4
Specialty (except psychiatric and substance abuse) hospitals	5.4	6.2	5.4	7.3	11.2	8.3	7.9	12.8	8.4	7.2	12.7
Nursing care facilities (skilled nursing facilities)	11.4	12.6	13.6	15.8	16.3	14.7	15.6	14.9	14.8	16.4	14.5
Residential intellectual and developmental disability, mental health, and substance abuse facilities	39.5	40.7	52.3	34.9	42.4	37.5	52.1	41.7	44.4	41.3	46.2
Continuing care retirement communities and assisted living facilities for the elderly	8.2	10.2	8.6	7.2	10.0	8.4	9.0	8.5	10.0	13.7	10.5
Other residential care facilities	35.2	31.8	40.6	39.9	43.8	63.0	40.2	61.0	59.4	34.8	45.8
Individual and family services	5.5	9.6	7.3	10.2	9.6	15.2	9.9	14.7	9.1	7.2	10.2
Community food and housing, and emergency and other relief services	3.0	6.1	4.9		4.2	4.2	15.7	7.5	7.2	9.5	11.0
Vocational rehabilitation services	10.8	9.0	11.3	20.8	14.1	18.0	12.2	17.0	19.1	11.6	12.2
Child day care services	3.9		2.4	6.5	1.6	2.9	0.6	7.8	5.8	0.7	2.9

### Data analysis

First, we described the rates of injuries from intentional WPV incidents across all of the health care facility types and then described the rates of change for each individual facility type. Next, we calculated average annual rates of WPV by occupation and industry (between 2011 and 2021/2022) across all occupations and industries. To assess whether annual averages were sensitive to outlier occupations or industries, we also calculated average annual rates of WPV without occupations or industries that fell above the 95th percentile in WPV rates. To assess whether year-to-year changes among occupations or industries were statistically significant, we used linear mixed-effects modeling. One set of models focused on annual rates of WPV (level 1) nested within occupations (level 2). A second set of models focused on annual rates of WPV (level 1) nested within industries (level 2). Models included data between 2011 and 2020; the 2021/2022 data were excluded as these data were reported biennially rather than annually.

For each of the 2 model sets, we specified models in the following stepwise manner: (1) intercept-only model, (2) model with time added as a level-1 predictor, and (3) model with a random slope specified for the time predictor. To assess whether multilevel models were sensitive to outlier occupations or industries, we also estimated these models without occupations or industries that fell above the 95th percentile in WPV rates. Model deviance values were assessed to determine whether changes to models yielded general improvement in model fit, where lower deviance values suggest a better fitting model.^19^ In the context of multilevel model-building, we also tested models in which nonlinear time effects were estimated. For both occupations and industries, the nonlinear time parameter was nonsignificant. As a result, we focused on models with linear change assumed.

## Results

Table 1 presents the rate of WPV incidents that resulted in at least 1 DAFW that occurred within different types of health care industries between 2011 and 2021/2022. (Appendix S1 shows WPV incidents per 10,000 workers by detailed occupation.) Rates are presented as the number of incidents per 10,000 full-time workers. Average rates of WPV per 10,000 widely varied across industries over the study period. More than 38% (*n* = 7) of industries/facility types had an average of 10 or more incidents per 10 000 full-time workers. Psychiatric and substance abuse hospitals; residential intellectual and developmental disability, mental health, and substance abuse facilities; and other residential care facilities had more than 43 WPV incidents per 10,000 full-time workers, on average. Some health care facility types had high, but stable rates of WPV between 2011 and 2021/2022, while others had rates that doubled over the study period. For example, WPV incidents at general medical and surgical hospitals increased 158% between 2011 and 2021/2022, while rates at nursing care facilities only increased 11% over the same period. Yet, on average, there were more incidents of WPV at nursing care facilities across the study period (15 per 10,000) as compared with general and medical surgical hospitals (8 per 10,000).

Table 2 shows average annual rates of WPV over time with respect to all industries and occupations analyzed. The table includes estimates with and without outliers. Outliers were defined based on having rates outside the 95th percentile of the average rates across occupations and industries. Using this measure, 3 occupations were considered outliers, psychiatric technicians, psychiatric aides, and occupational therapy aides. One industry type, psychiatric and substance use hospitals, was considered an outlier. Average annual rates of WPV were significantly larger before excluding outlier occupations and industries. For industries, average annual WPV rates ranged from 1.3 times higher before excluding outliers in 2013 to nearly twice as high in 2013, 2017, and 2018. Differences in average annual WPV rates for occupations were even more striking, ranging from 2.4 times greater in 2017 to 4.7 times larger in 2013 before excluding outliers.

**Table 2.** Average annual rates of workplace violence over time across health care occupations and industries.

	Occupation		Industry	
Year^a^	Outliers removed^b^	All	Outliers removed^b^	All
2011	9.4	23.8	9.7	13.4
2012	10.2	24.5	10.7	14.4
2013	7.3	34.7	8.0	15.6
2014	9.1	35.8	11.7	17.8
2015	10.6	34.1	11.5	16.1
2016	9.8	28.4	9.2	17.1
2017	11.3	25.3	9.8	18.9
2018	8.5	29.9	10.4	20.7
2019	7.0	32.1	12.0	20.4
2020	10.1	30.5	11.6	17.6
2021–2022^c^	7.2	29.0	12.6	18.4

^a^Annual estimates represent average rates across units (eg, occupation, industry).

^b^Cases with rates >50 were treated as outliers; a rate of 50 was around the 95th percentile in the overall distribution of rates across cases.

^c^Estimate represents the average 2021–2022 biannual rate across units.

Volatility in yearly estimates makes discerning trends difficult, but WPV rates appeared to generally increase over time. For industries, there was an average rate of 13.4 in 2011 and an average rate of 18.4 in 2021/2022. For occupations, there was an average rate of 23.8 in 2011 to an average rate of 29.0 in 2021/2022. After excluding outlier cases, trends over time for occupations appeared more flat, with an average rate of 9.4 in 2011 and an average rate of 10.1 in 2020 (and 7.2 in 2021/2022). An apparent upward trend remained for industries, however, when outliers were excluded, with an average rate of 9.7 in 2011 and an average rate of 11.6 in 2020 (and 12.6 in 2021/2022).

Table 3 displays results, both with and without outliers, from longitudinal multilevel modeling and associated annual rate estimation for both occupations and industries. The model-building process for occupations, when outliers were included, yielded the lowest model deviance value for the model in which a random slope for time was specified. Although the association between time (in year units) and annual rate of WPV was nonsignificant (*b* = 1.14, *P* = .30), the association between time and annual rate of WPV varied significantly across occupations (variance = 12.40). When outlier occupations were excluded, model deviance values were equivalent for models with and without a random slope parameter for time. Thus, we favored the more parsimonious model (ie, the model with fewer parameter estimates), and removed the random slope for time from the final model. Results from this model also yielded a nonsignificant association between time (in year units) and annual rates of WPV (*b* = 0.16, *P* = .26). Given the absence of a significant random slope for time, this nonsignificant association appeared to be consistent across all non-outlier occupations. Notably, the models without outlier occupations yielded appreciably lower model deviance values relative to the models that retained outliers, suggesting that the removal of outliers improved model fit.

**Table 3.** Estimated rates of workplace violence over time across health care occupations and industries.

	Occupation		Industry	
Yearb^b^	Outliers removed^a^	All	Outliers removed^a^	All
2011	8.8	20.1	9.3	13.0
2012	9.0	21.2	9.7	13.7
2013	9.2	22.4	10.0	14.4
2014	9.3	23.5	10.4	15.1
2015	9.5	24.6	10.7	15.8
2016	9.6	25.8	11.0	16.5
2017	9.8	26.9	11.4	17.2
2018	9.9	28.1	11.7	17.9
2019	10.1	29.2	12.1	18.6
2020	10.2	30.3	12.4	19.3
2021–2022^c^	7.2	29.0	12.6	18.4
Average annual rate of change from 2011 to 2020	0.16 (ns; *P* = .26)	1.14 (ns; *P* = .30)	0.34***	0.71*
No. of units	42	45	17	18
No. of observations	189	210	147	162

*P < .05; ***P < .001; ns = nonsignificant (*P* > .05).

^a^Cases with rates >50 were treated as outliers; a rate of 50 was around the 95th percentile in the overall distribution of rates across cases.

^b^Annual estimates were derived from linear mixed-effects models, with annual rates nested in units (eg, occupation, industry).

^c^Estimate represents the average 2021/2022 biannual rate across units.

Similar to occupations, the model-building process for industries, when outliers were included, yielded the lowest model deviance value for the model in which a random slope for time was specified. Results from this model indicated that time (in year units) was significantly associated with annual rates of WPV among industries (*b* = 0.71, *P* = .03). On average, annual rates of WPV increased by 0.71 units per year across industries. However, this rate of change had significant variance across industries (variance = 1.58). When outlier industries were excluded, model deviance values were equivalent for models with and without a random slope parameter for time. Thus, similar to the occupation models, we favored the more parsimonious model (ie, the model with fewer parameter estimates), and removed the random slope for time from the final model. Results from this model retained a significant and positive association between time (in year units) and annual rates of WPV (*b* = 0.34, *P* < .001). In the absence of outlier industries, annual rates of WPV increased by 0.34 units per year. This association did not differ significantly across industries (ie, nonsignificant random slope parameter for time). The models with outlier industries excluded also yielded notably lower model deviance values relative to models that retained outliers, suggesting that the removal of outliers improved model fit.

## Discussion

Overall, this study's findings suggest that WPV within health care industries is significantly increasing over time, yet across all health care occupations there was no statistically significant increase in average rates of WPV, particularly when excluding outlier occupations. Previously reported average annual rates of WPV often do not exclude significant outliers within occupations and facilities—which drive high rates of WPV reported and require focused interventions. However, data suppression and year-over-year volatility make it hard to discern time trends for WPV. It is also notable that the upward trend of WPV observed in health industries occurred prior to the COVID-19 pandemic. The rate of WPV in 2021/2022 was lower than the previous year for health care occupations, suggesting that, although the pandemic increased stress within health care settings, there may be other contributing factors towards the rising rates of WPV.

Rates of WPV across health care facility types increased by nearly 30% between 2011 and 2021/2022 (when excluding outliers). Earlier studies have typically focused on WPV within single health care occupations or settings. However, recognizing that WPV is rising across all health care settings is crucial information because interventions are often targeted at the organizational or facility level.

States, hospital systems, and professional organizations are quickly working to address WPV through sharing information on policies and interventions to protect health care workers and mitigate violence. National organizations like the American Hospital Association and the Joint Commission have updated prevention standards and disseminated effective strategies for WPV prevention and remediation for health systems to draw upon.^23,26-28^ While addressing the immediate crisis of WPV is essential, it may overlook a broader issue—that WPV was escalating even before the pandemic began. This trend suggests that systemic factors are contributing to WPV and should be considered in efforts to protect health care workers. Additionally, understanding variations in WPV across different types of facilities and occupations (eg, nurses in psychiatric hospitals) and by state can enhance efforts to detect and target effective interventions and policies to support the health care workforce. Evaluating the impact of targeted interventions, including hospital-based prevention strategies and state legislation, will be critical in reducing WPV over time.

Previous work that has explored trends in WPV across multiple facilities or occupation types did not examine the influence of outliers that may be driving the high rates of WPV in the United States. For example, in Hawkins and Ghaziri,^20^ although higher rates of WPV were noted for several groups of occupations, including nurse, psychiatric, and home health aides, the analyses did not exclude outlying health care occupations. Our study differs from prior research by identifying and excluding outliers from our longitudinal analyses of WPV rates. By comparing rates with and without outliers, we found that removing 3 outlier occupation types resulted in reducing the WPV average from 29 to 7 incidents per 10,000 workers in 2021/2022, suggesting that previous high rates were likely driven by a few occupations with exceptionally high WPV occurrences. It is not a new finding that some occupations and settings are at a higher risk of WPV,^21,22^ suggesting that future work should examine WPV accounting for nuances within and across health care. However, WPV is not to be “expected” and a focus on addressing rates of WPV within the “outlier” occupations and industries is critical and will require targeted interventions.

Many states have enacted legislation to ameliorate WPV.^23^ However, it is unclear if state policies on WPV address the nuances of varied rates of WPV across facilities and occupations. A recent article summarizing state policies suggests that the majority of state WPV laws are focused on increasing the penalty for those who perpetrate WPV.^23^ It is unclear if there is evidence to support legislation that aims to enact penalties for WPV. Although the vast majority of states have laws that increase the punishment for violence towards health care workers, rates found in this study and others suggest that WPV is commonly occurring and intractable despite these laws.

Federal legislation to address WPV has failed to advance in Congress, leaving the role of addressing WPV at the state level. Some states have begun to enact policies that require health care systems to implement prevention strategies to reduce WPV. For example, Minnesota passed legislation in 2022 that requires hospitals to document action plans for WPV, including systematically reviewing incidents, requiring staff training, and creating procedures to allow health workers to request additional staffing to prevent WPV.^24^ This study did not observe within-state variation in WPV and future work would benefit from examining how state WPV laws and policies impact rates of WPV for health care workers across health care settings.

This study did not assess differences in WPV by sociodemographic characteristics of health care workers, which may have revealed other factors that contribute to rates of WPV. Previous work has found that women and people from racially and ethnically minoritized backgrounds may be more likely to experience WPV.^19^ This study did observe that many occupations that employ predominantly people of color had higher rates of WPV than occupations that proportionally have fewer people of color in their workforce.^25^ This finding could suggest that there is an aspect of the type of work that may be associated with rates of WPV across sociodemographic characteristics. More work needs to be conducted to examine if existing WPV strategies and interventions reduce disproportionate violence within settings and direct care and psychiatric occupations.

A limitation of the current study is that WPV incidents were restricted to those that resulted in a day away from work due to injury. This likely far underrepresented WPV rates since incidents of WPV that did not result in a DAFW were not described in the present study. Verbal aggression and intimidation towards health care workers, which also negatively impact health care worker well-being, were not observed in the current study.^3,7,14^ This study utilized employer-reported information, requiring a health care worker to have notified their employer of the event to be accounted for in the data source. There is evidence that health care workers underreport incidents of WPV.^7,13^ As such, the rates of WPV are likely even higher than what are presented in the analysis. Although our study examined WPV rates that are annualized per 10,000 full-time workers, which accounts for workforce size differences across occupations and industries, future work could examine the implications of weighting the size of the work—as there are far more opportunities for WPV events to occur within larger workforces. Missing data due to suppression and data volatility across study years make trends difficult to discern. Unfortunately, there are no other nationally representative data sources that present data by occupation and industry/facility type that would offer alternatives to the SOII. This significantly limits the ability of researchers and policymakers to understand the scope of WPV, evaluate the effectiveness of WPV policies, and target interventions.

## Supplementary Material

qxae134_Supplementary_Data

## Data Availability

This study used data drawn from the BLS’ Survey of Occupational Injuries and Illness (SOII). The SOII contains employer-reported information on illnesses and injuries that occur within an employment-based setting in the United States. The SOII is a survey of approximately 200, 000 employers annually. The SOII data are publicly available and annualized per 10,000 full-time workers.
