# Supplementary material for: Trends in workplace violence for health care occupations and facilities over the last 10 years
Source: Health Aff Sch. 2024 Oct 23;2(12):qxae134. doi: 10.1093/haschl/qxae134 (PMC11630250; doi:10.1093/haschl/qxae134)
Supplement: qxae134_Supplementary_Data [file qxae134_supplementary_data.zip › Appendix 1.WPV.docx]

Appendix 1. Rate of WPV Incidents per 10,000 Full Time Workers within Included Health Care Occupations (2011-2021/22)

| **Selected Healthcare Occupations** | **2011** | **2012** | **2013** | **2014** | **2015** | **2016** | **2017** | **2018** | **2019** | **2020** | **2021/**  **2022** |
| --- | --- | --- | --- | --- | --- | --- | --- | --- | --- | --- | --- |
| Clinical, counseling, and school psychologists |  |  |  |  |  |  |  | 5.5 | 7.5 | 5.5 |  |
| Substance abuse and behavioral disorder counselors | 6.6 | 20.8 | 4.5 | 6.8 | 12.6 | 9.1 |  |  |  |  |  |
| Educational, guidance, and career counselors and advisors |  | 3 |  | 4.8 | 6.3 | 7.7 | 10.4 | 6.3 |  | 5.4 | 12.4 |
| Marriage and family therapists | 8.5 |  |  |  |  |  |  | 8.2 |  |  |  |
| Mental health counselors | 29.6 | 22.6 | 28.5 | 28.3 | 31.3 | 30.4 |  |  |  |  |  |
| Rehabilitation counselors | 12.2 | 7 | 7.7 | 8.7 | 43.1 | 24.1 | 7.5 | 4 | 7.8 | 5.1 | 15.9 |
| Substance abuse, behavioral disorder, and mental health counselors |  |  |  |  |  |  |  |  | 10.9 | 22.1 |  |
| Child, family, and school social workers | 3.4 | 2.2 | 5.1 | 3.8 | 8.9 | 3.9 | 4.5 | 7 | 9.4 | 4.3 | 4.8 |
| Healthcare social workers |  | 3.1 | 2.3 | 1.6 | 1.7 | 1.6 | 5.3 |  |  | 3.1 |  |
| Mental health and substance abuse social workers | 5.9 | 2.7 |  | 6.1 | 6.9 | 9.7 | 3.5 |  | 3.8 | 4.3 | 13.7 |
| Social and human service assistants | 3 | 14.5 | 8.9 | 8.6 | 6.8 | 9.6 | 12.3 | 8.9 | 7.2 | 4.4 | 5.6 |
| Community health workers |  |  |  |  |  |  |  |  |  | 15.9 | 2.5 |
| Psychiatrists |  |  |  | 12 |  |  |  |  |  |  |  |
| Physician assistants | 2.7 |  |  |  |  |  |  |  |  |  |  |
| Occupational therapists |  |  |  |  | 3.4 | 10.4 |  | 4.2 | 4.6 |  | 4.7 |
| Physical therapists |  |  |  | 1.2 | 1.5 |  |  |  | 4.2 | 1.5 | 4.1 |
| Recreational therapists | 41 | 27.5 |  | 23.6 | 29.8 |  | 24.5 | 16.3 |  | 39.2 |  |
| Respiratory therapists |  |  | 3.6 | 3.1 | 2.6 | 1.9 | 3.3 | 11.5 | 2.4 |  | 3.5 |
| Speech-language pathologists |  |  |  |  | 6.8 |  | 11.3 | 22.2 | 2.4 |  | 6.1 |
| Registered nurses | 5.8 | 5.9 | 6.5 | 7.5 | 6.3 | 7.8 | 8.8 | 8.7 | 9.3 | 14.2 | 11.9 |
| Nurse practitioners |  |  |  |  |  |  |  |  | 3 | 1.8 | 0.6 |
| Physicians, all other; and ophthalmologists, except pediatric |  |  |  |  |  |  |  |  | 0.5 |  |  |
| Medical and clinical laboratory technicians | 4.7 |  | 1.5 | 10.5 |  | 4.3 |  |  |  |  |  |
| Cardiovascular technologists and technicians |  |  |  |  |  |  | 3.8 | 3.3 |  |  | 4.4 |
| Diagnostic medical sonographers |  |  |  |  |  |  |  |  |  | 3.6 | 2.1 |
| Radiologic technologists and technicians |  |  |  |  | 1.8 |  |  | 2.2 |  | 3.2 | 1.7 |
| Emergency medical technicians and paramedics | 5.2 | 4.6 | 4 | 7.4 | 7.2 | 7.1 | 7.6 | 8.9 |  |  |  |
| Dietetic technicians |  |  |  |  |  | 7.7 |  |  |  |  |  |
| Pharmacy technicians |  |  | 1.7 |  |  |  |  |  |  |  | 0.4 |
| Psychiatric technicians | 157.7 | 173.1 | 122.6 | 206.8 | 236.9 | 233.7 |  | 275.3 | 316.3 | 254.1 | 215 |
| Surgical technologists |  |  | 2.5 |  |  |  |  |  |  |  | 1.1 |
| Licensed practical and licensed vocational nurses | 7.7 | 8 | 6.7 | 7.7 | 7 | 8.7 | 5.8 | 7.1 | 11.6 | 12.2 | 8.9 |
| Health information technologists, medical registrars, surgical assistants, and healthcare practitioners and technical workers, all other |  |  |  |  |  |  |  |  | 19.9 |  |  |
| Home health aides | 4.7 | 3.8 | 6 | 6.2 | 6.3 | 6.9 | 9.8 | 5.4 |  |  |  |
| Psychiatric aides | 105.7 | 89.9 | 439.5 | 426.4 | 221.3 | 195.3 | 307 | 190.8 |  |  |  |
| Nursing assistants |  | 23.5 | 26.6 | 28.4 | 28.4 | 26.1 | 29 | 26.3 |  |  |  |
| Orderlies |  |  | 11 | 8.9 |  | 10.5 | 6.2 |  |  |  |  |
| Nursing assistants |  |  |  |  |  |  |  |  | 29.9 | 38.3 | 33.6 |
| Orderlies |  |  |  |  |  |  |  |  |  | 9.2 |  |
| Psychiatric aides |  |  |  |  |  |  |  |  | 178.9 | 254.3 | 389 |
| Occupational therapy assistants |  |  |  |  |  |  | 8.8 |  | 8.1 | 5.7 |  |
| Occupational therapy aides |  |  |  |  | 137.9 |  | 37.8 |  | 51.7 |  |  |
| Physical therapist assistants |  |  |  |  |  | 4.4 |  |  |  |  |  |
| Medical assistants | 0.6 |  | 1.4 | 0.6 | 1.6 |  | 3.8 | 2.1 | 1.1 | 1.4 | 2.3 |
| Phlebotomists |  | 3.7 | 2.8 | 5.5 | 2.4 | 3.4 | 21.3 | 3.9 | 3 | 2.2 | 6.8 |
